# The Effect of Photodynamic Therapy on Oral-Premalignant Lesions: A Systematic Review 

**DOI:** 10.4317/jced.59348

**Published:** 2022-03-01

**Authors:** Ruchika Choudhary, Sujatha S. Reddy, Ravleen Nagi, Rakesh Nagaraju, Sreekanth P. Kunjumon, Ritu Sen

**Affiliations:** 1 Assistant Professor. Department of Oral Medicine and Radiology, NIMS Dental College and Hospital, Jaipur, Rajasthan; 2 Professor. Department of Oral Medicine and Radiology, Faculty of Dental Sciences, MS Ramaiah University of Applied Sciences, MSRIT post Mathikere, 560054, Bangalore, India; 3 Reader. Department of Oral Medicine and Radiology, Saveetha Dental College, 600077, Chennai, Tamil Nadu; 4 Professor and Head. Department of Oral Medicine and Radiology, Faculty of Dental Sciences, MS Ramaiah University of Applied Sciences, MSRIT post Mathikere, 560054, Bangalore, India; 5 Post Graduate Student. Department of Oral Medicine and Radiology, Faculty of Dental Sciences, MS Ramaiah University of Applied Sciences, MSRIT post Mathikere, 560054, Bangalore, India; 6 Post Graduate Student. Department of Oral Medicine and Radiology, Faculty of Dental Sciences, MS Ramaiah University of Applied Sciences, MSRIT post Mathikere, 560054, Bangalore, India

## Abstract

**Background:**

Dentists now have access to a wide range of unique treatment methods as a result of substantial scientific and technological breakthroughs in the field of dentistry. Photodynamic therapy (PDT) is a non-invasive treatment procedure that use photosensitizers, a specific wavelength of light, and the production of singlet oxygen and reactive oxygen species (ROS) to kill undesired eukaryotic cells (such as oral tumors) and harmful microbes. In several disciplines of dentistry, it is seen as a valid therapeutic option. The purpose of this study was to examine the effectiveness and side effects of PDT in the treatment of oral premalignant lesions.

**Material and Methods:**

Three search engines (PubMed, ISI Web of Science, and the Cochrane Library) were used to conduct a systematic review using the phrases photodynamic therapy and PDT in combination with other terms. To define our study eligibility criteria, we used the Population, Intervention and Comparison, Outcomes, and Study design technique.

**Results:**

Initial results were 33. Definitely, 18 studies met our selection criteria.

**Conclusions:**

Our analysis suggests ALA- PDT as a promising therapeutic modality for OEL lesions which should be treated first with the topical ALA-PDT using either the LED or laser light for successful clinical outcome for OEL lesions.

** Key words:**Photodynamic Therapy, Photosensitizer, Aminolevulinic Acid (ALA), Methylene Blue (MB), Toludine Blue, Oral Leukoplakia, Oral Erythroplakia, Oral Verrucous hyperplasia, Oral Lichen Planus.

## Introduction

A variety of therapeutic approaches have been proposed for the treatment of oral per-malignant lesions (such as leukoplakia [OL], oral erythroplakia [OE], oral verrucous hyperplasia [OVH] and oral lichen planus [OLP]). Some of the treatment options include topical medication (such as vitamin A, antibiotics, and steroids), LASER ablation, cryotherapy, and surgical excision. Non-surgical treatment approaches for pre-malignant lesions (such as topical medication delivery) may be beneficial in the short term, but they have been linked to high recurrence rates. Furthermore, it has been demonstrated that surgical treatment of oral precancerous lesions increases morbidity and scar tissue development.

For the treatment of premalignant lesions in the mouth, photodynamic therapy (PDT) is a novel treatment option. PDT includes the interaction of a light source with a chemical dye or photosensitizer in the presence of oxygen (PS). As a result of this interaction, reactive oxygen species are formed, causing oxidative damage to microbial cell walls as well as pre-malignant and malignant cells. Oscar Raab discovered in 1900 that paramecia was destroyed by the interaction of acridine (a dye) and visible light in the presence of oxygen [32]. Later, Niels Finsen undertook research on the use of the arc light in phototherapy, for which he was awarded the Nobel Prize in 1903 [32]. Professor Hermann von Tappeiner, head of the Pharmacological Institute of the Ludwig-Maximilians University in Munich [33], coined the phrase ‘photodynamic action’ (‘photodynamische Wirkung’) in 1904. In 1913, the German physician Friedrich Meyer-Betz conducted the first investigation with porphyrins, which was first dubbed photo radiation therapy (PRT). On his own skin, he evaluated the effects of hematoporphyrin-PRT. With early clinical argon dye LASER, John Toth, as product manager for Cooper Medical Devices Corp, Cooper Lasersonics, recognised the photodynamic chemical impact of the therapy and authored the first white paper renaming the therapy as Photodynamic Therapy (PDT). This was done to help fund the establishment of ten clinical locations in Japan, where the word radiation was associated with negative connotations [32]. The University of Berlin’s Auler and Banzer identified the characteristic red fluorescence of porphyrins in mouse tumours in 1942. This discovery marked the start of the field of photodynamic diagnostics (PDD).

PDT was used to treat 10 individuals with oral lichen planus (OLP) in a research by Rakesh *et al*. (1). The results demonstrated that OLP was completely eradicated, with no recurrence during a four-year follow-up period. Mirza reported similar findings. S *et al*. (17). According to the findings of these studies, PDT may be a potential therapy approach for the management of oral premalignant lesions (1-17). PDT treatment for oral premalignant lesions, however, resulted in recurrence and secondary infection in three individuals after six months, according to research (16). In this context, there appears to be considerable disagreement over the effectiveness of PDT in the treatment of oral premalignant lesions. The goal of this study was to look at the efficacy and side effects of PDT in the treatment of oral premalignant lesions in a systematic way.

## Material and Methods

-Focused Questions

1. The Preferred Reporting Items for Systematic Reviews and Meta-Analyses (PRISMA) criteria were utilised to construct a focused query. The following was the question that was addressed: -

2. Is PDT useful in the treatment of premalignant lesions in the oral cavity?

3. Which are the photosensitizer most commonly used?

4. Is PDT cause any adverse effect during or post-treatment?

5. Follow-up and Recurrence Rate?

-Eligibility Criteria

The following requirements had to be met:

Original investigations, clinical studies, case reports, and intervention studies were all required. -the efficacy of PDT in the treatment of oral premalignant lesions, and works written solely in English.

Review papers, experimental research, letters to the editor, and unpublished works were not taken into account.

Search Strategy

**Figure 1:**
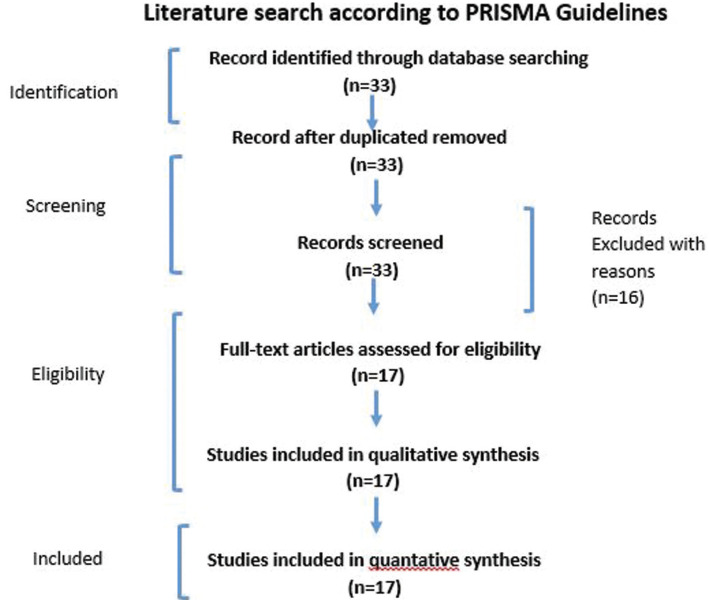
Flow chart shows study selection criteria based on Preferred Reporting Items for Systematic Reviews (PRISMA) guidelines.

In PubMed/Medline (National Library of Medicine, Bethesda, Maryland), Google Scholar, EMBASE, and the ISI Web of Knowledge database, the following keywords were searched from 2000 to 2020: Oral premalignant lesions, oral lichen planus, and photodynamic treatment are all conditions that can be treated using photodynamic therapy. The writers reviewed and double-checked the titles and abstracts of articles that satisfied the qualifying requirements. The entire texts of eligible articles were checked and independently appraised using the title and abstract eligibility criteria. Following that, the authors discussed and agreed on the reference lists of original and review studies that they thought were significant (Fig. 1). In the initial search, 33 studies were discovered. In total, 17 studies (1,2,4,5,8-13,15-17,23,24) were included and data was extracted from them. The current study’s design was tailored to primarily summarize the important data.

## Results

-General characteristics of the studies

Pre-existing oral premalignant lesions with or without dysplasia that were clinically and histopathologically identified were included in all investigations (1,2,4,5,8-13,15-17,23,24). OL, OEL, OLP, and OVH were seen in all premalignant lesions treated in all studies (1,2,4,5,8-13,15-17,23,24). 297 instances of premalignant lesions were documented in this study (1,2,4,5,8-13,15-17,23,24). The buccal and labial mucosa (maxilla and mandible), tongue, palate, floor of the mouth, bucco-gingival sulcus, and concomitant gingiva were characterized in fifteen investigations (1,4,5,8-13,15-17,23,24,26).

In a study by Koty Naik *et al*., the effectiveness of PDT was compared to that of conventional treatment (control group) in the management of premalignant lesions (12). (The OL and OLP acronyms stand for old and new and old In the research, PDT was compared to LLT and topical corticosteroids (17). Chuan *et al*. conducted a clinical evaluation of OLE and PDT utilising either light-emitting diode or laser light (26).

-Included research’ LASER-related parameters

Diode LASERS, light emitting diode (LED), and dye LASERS were used in teen (1,2,5,12,13,15,16,17,26,28), three (4,8,10) and two (24,11) studies, respectively. Diode LASER (24,11), argon pumped dye (24) and pulsed dye LASER (11) were used in these research. In one research (23) xenon arch lamp light was used. All of the studies used LASERS with wavelengths, energy fluences, and power densities ranging from 480 to 670 nanometers (nm), 6 to 200 joules per square centimetre (J/cm2), and 100 to 150 milliwatts per square centimetre (mW/cm2) (1,2,4,5,8-13,15-17,23,24). Thirteen studies (1,4,5,9-13,16,17,23,24,26) reported a number of sessions ranging from one to twelve, ( Table 1,  Table 1 cont.,  Table 1 cont.-1.).

**Table 1:**
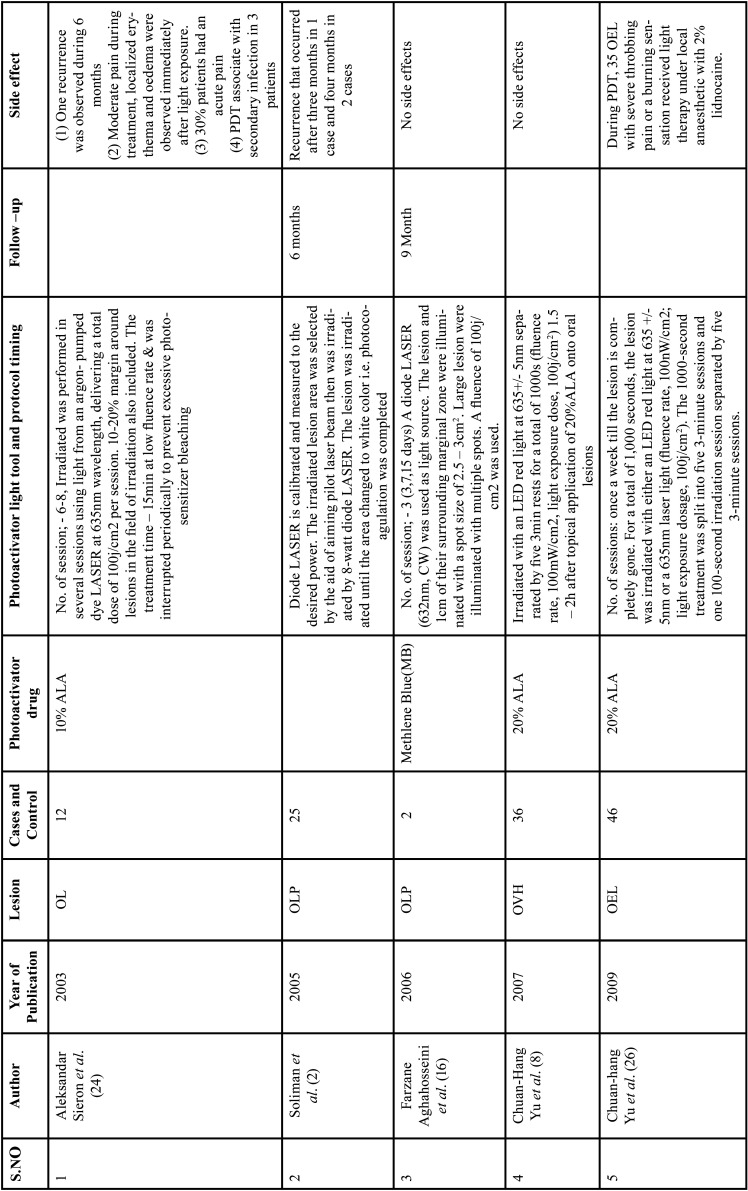
Characteristics of included studies based on Population, Intervention, Comparison and Outcome (PICO) model.

**Table 1 cont.**
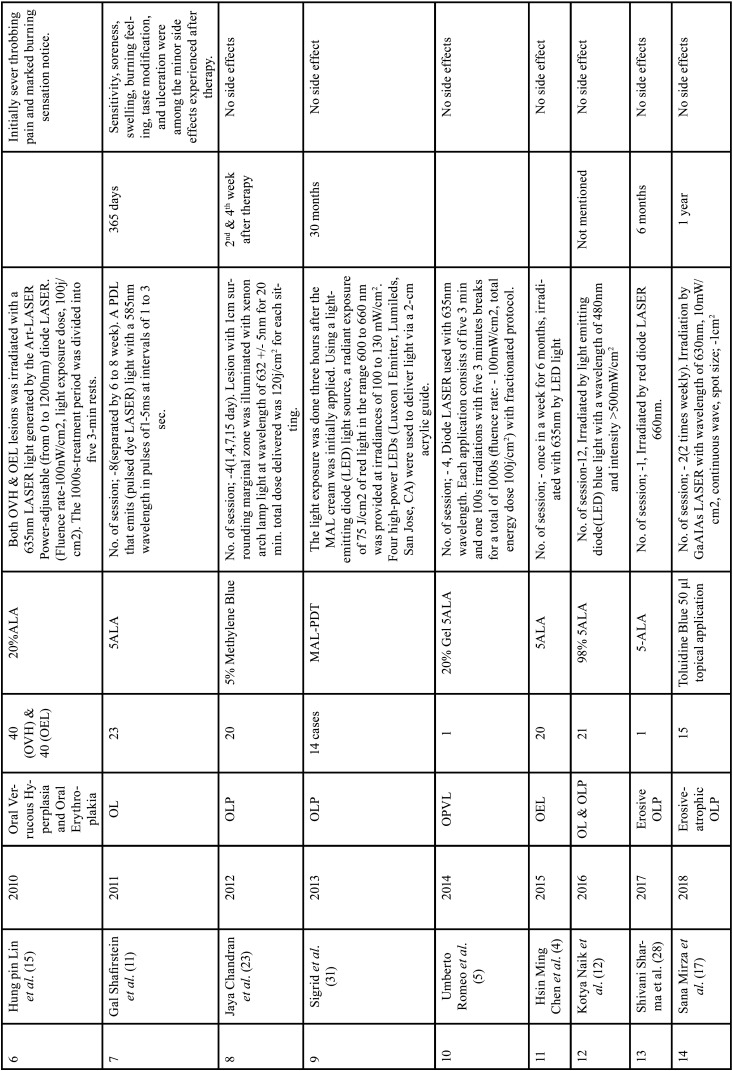
Characteristics of included studies based on Population, Intervention, Comparison and Outcome (PICO) model.

**Table 1 cont.-1:**
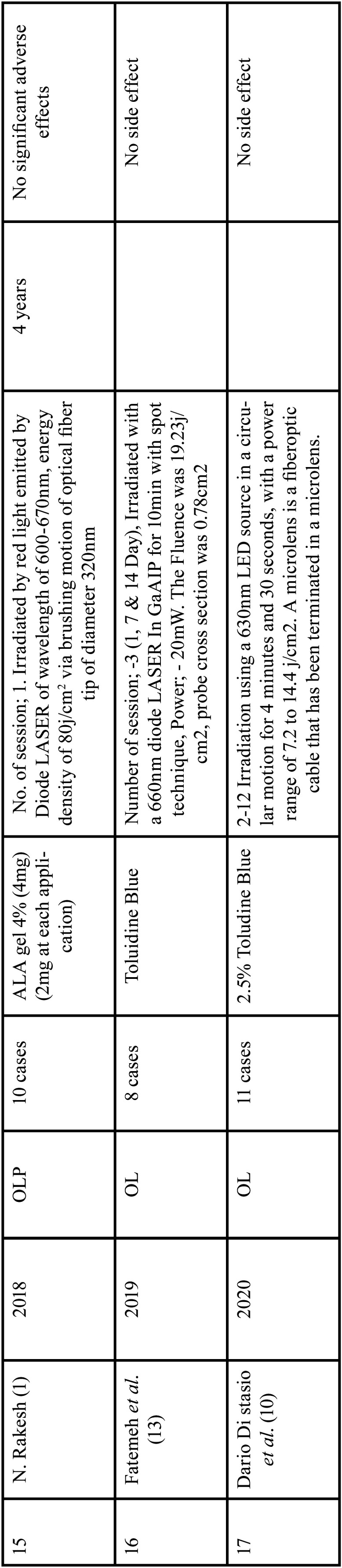
Characteristics of included studies based on Population, Intervention, Comparison and Outcome (PICO) model.

## Discussion

In 2020, Dario *et al*. [10] treated 11 OL with PDT; the reported result revealed that 40% of the lesion had a complete response(CR), and 46.7% reported a partial response(PR), while in 13.4% no response(NR) at the end of the treatment and most commonly involved site was the adherent gingiva. In 2007, Chen et.al., treated 32 OL lesions with topical ALA with PDT; the reported results revealed that 1/3 of the 32 OL lesion could achieve a CR after treatment twice a week. Even after 10 months of treatment, 94 percent (16/17) of PR or NR OL lesions were challenging to attain a CR. During the follow-up period, 7.8% (28/36) of the follow-up OL lesions showed a small increase in the size of the residual lesion [8]. In 2006, Aghahosseini *et al*., reported 5 OLP lesion, treated with MB mediated PDT. Two lesions completely resolve (CR). A PR (more than 50% improvement) was observed in TWO other lesions. There was no recurrence in improved lesions after 9 months follow-up. But no improvement was observed in lesion on the tongue [16]. In study, Umber *et al*. (2014), lesion showed CR after 4 session of PDT and no recurrence was noted after 12 months [5]. Koty Naik *et al*. (2016) studied 13 patients with 24 OL lesions and 8 patients with 29 OLP lesions who were treated with PDT (study group) and conventional therapy (control group). According to the distribution of OL and OLP lesions for PDT, 9 (75.0%) lesions were on the BM, 2 (16.66%) on the tongue, and 1 (8.33%) lesion on the associated gingiva in OL, and 9 (90.0%) lesions were on the buccal mucosa, 1 (10.0%) lesion on the attached gingiva in LP. Although 10 (83.33%) lesions were on the BM, 1 (8.33%) lesion on the tongue, and 1 (8.33%) lesion on the gingiva in conventional therapy, in OLP, 6 (60.0%) lesions were on the BM, 2 (20.0%) on the connected gingiva, and 2 (20.0%) on the tongue. In the OLP PDT group, two (16.66 percent) of the 12 lesions were CR, eight (66.66 percent) were PR, and two (16.66 percent) were NR. Twelve lesions were found in the conventional group, with two (16.66%) indicating PR and nine (75.0%) showing NR. Twelve lesions were found in the conventional group, with two (16.66%) indicating PR and nine (75.0%) showing NR. In OLP with PDT, 8 (80.0%) of the 10 lesions diagnosed had PR and 2 (20.0%) had NR, whereas in conventional treatment, 10 lesions were found, with 8 (80.0%) having PR and 2 (20.0%) having NR [16].

In 2007, Chen *et al*.; treated 24 OVH lesions, 65 OL lesions and 6 OEL lesions with topical ALA-PDT once a week and 32 OL lesions were treated with ALA-PDT twice a week. After 1-6 treatments, all 24 OVH lesions showed CR, while 65 OL lesions showed CR in 5, PR in 33, and NR in 27. In the 32 OL lesions treated twice a week with ALA PDT, 11 showed CR and 21 showed PR. The clinical result of the 32 OL lesions treated twice a week was significantly better than the 65 OL lesions treated once a week. According to this study, CR of an OVH lesion can be accomplished in as little as seven treatments in a week. OL lesions treated twice a week have a significant better clinical outcome than OL lesions treated once a week. OEL lesions treated once a week have a significantly better clinical outcome than OL lesions treated once a week [8]. In 2011, Galll Shafirstein *et al*.; reported 23 patients of OL aged between 37 to 79 years, treated with 5-ALA and pulsed dye laser, noted that more than 75% resolution of the lesion; PR, reduction of at least 25% and NR, reduction by less than 25% [11]. In a study reported in 2019, Fatemeh *et al*.; treated 11 patient of OLP lesion by comparing the effect of PDT (toludine blue) and topical corticosteroid (0.1% triamcinolone acetonide). PDT alone and in comparison with the control side without any interventional up to session 3/day 21 was significantly more effective, but by starting the topical corticosteroid therapy for both side of the intervention and control, this discrepancy was compensated by 4-week application of topical corticosteroid. Author state that PDT can reduce the treatment session, while topical corticosteroids application may need to be continued for several weeks. Also, PDT can be used as an adjuvant therapy in combination with topical steroid for resistant or recurrent OLP [13]. Hung Pin *et al*. (2010) reported 40 OVH and 40 OEL treated with ALA-PDT, finding that all 40 OEL lesions displayed CR after an average of 3.6 PDT therapy, with 38 showing CR and 2 showing PR after an average of 3.4 PDT treatment [13]. In 2018, study Mirza Sana *et al*.; reported 25 OLP lesion on the tongue or buccal mucosa treated with toluidine blue PDT, LLT and topical corticosteroids application, they indicated that PDT and LLT both are effective in treatment of Erosive-atrophic form OLP. However, PDT showed superior results than LLT, while corticosteroid application reduced significantly greater pain as compared to both PDT and LLLT groups [17]. In 2018 study , Rakesh *et al*.; treated 10 patient Erosive OLP between the age group of 20-70 with ALA-PDT, reported excellent result in 1 session with remarkable reduction in sign and symptoms, although post-inflammatory hyperpigmentation persisted and no recurrence in 4 year follow-up [1]. Similarly Shivani *et al*. (2017)., treated Erosive OLP with ALA –PDT , showed excellent result in 1 session with no recurrence in 6 month follow-up [28]. Sadaksharam *et al*. (2012) reported 20 patients OLP treated with 5% Methylene Blue mediated PDT (xenon arc Lamp), noted that no improvement in 3 cases; moderate improvement in 9 cases; marked improvement in 6 cases and CR in two cases. Aleksander Sieron *et al*. (2003), reported a study, 12 patient with OL treated with 10%ALA using PDT (argon-pumped dye LASER). Out of 12 Patient, 10 showed CR, 2 – NR and 1 recurrence was observed during 6 months [24]. Yu-Chuan *et al*. (2009) investigated 46 OEL cases, 20 of which were treated with ALA-PDT using LED light and 26 of which were treated with ALA-PDT using LASER light of the same wavelength (635), and found that the 20 LED light-treated OEL lesions exhibited CR in 17 instances and PR in three cases. CR was found in 25 of the 26 laser-light-treated OELs, whereas PR was found in one [26]. Sigrid *et al*. (2013) described 14 instances of OLP where one side was treated with MAL-PDT and the other was not. All of the participants in the experiment had improved after three months. Two patients had relapsed by the 6-month follow-up, although new lesions were only found in areas where amalgam fillings were in direct contact. These patients were subsequently advised to have their fillings replaced, and as a consequence, the mucosa healed completely.

## Conclusions

Our findings point to ALA-PDT as a viable treatment option for dysplastic OEL lesions, which should be treated initially with topical ALA-PDT using either LED or laser light for a positive clinical result. Furthermore, topical ALA-PDT is a minimally invasive approach that may be utilized to treat recurring or resistant lesions on a regular basis without creating major short- or long-term negative effects. However, because there is insufficient evidence to support ALA-PDT as a first-line treatment for dysplastic OEL lesions, more longitudinal follow-up studies should be encouraged to demonstrate its therapeutic usefulness in this area. In addition, more comparative studies should be conducted to compare the effectiveness of PDT with LED or laser therapy and with other therapeutic modalities for dysplastic OEL lesions.
